# Determinants of adherence to wrap-around care in child and family services

**DOI:** 10.1186/s12913-018-3774-6

**Published:** 2019-01-28

**Authors:** Noortje M. Pannebakker, Margot A. H. Fleuren, Eline Vlasblom, Mattijs E. Numans, Sijmen A. Reijneveld, Paul L. Kocken

**Affiliations:** 1 Department of Public Health and Primary Care, Leiden University Medical Center, Leiden, The Netherlands; 2 Department Child Health, TNO, P.O. Box 3005, 2301 DA Leiden, The Netherlands; 3 Department of Clinical, Neuro- and developmental Psychology, Faculty of Behavioural and Movement Sciences, Amsterdam Public Health (APH) research institute, Free University Amsterdam, Van der Boechorststraat 7, 1081 BT Amsterdam, The Netherlands; 4 Department of Public Health and Primary Care and Leiden University Medical Center Campus The Hague, Leiden University Medical Center, P.O. Box 9600, 2300 RC Leiden, The Netherlands; 5 Department of Health Sciences, University of Groningen, University Medical Center Groningen, Antonius Deusinglaan 1, 9713 AV Groningen, The Netherlands

**Keywords:** Adherence, Wrap-around care, Innovation strategy

## Abstract

**Background:**

The aim of this study is to understand the determinants of adherence to wrap-around care (WAC) by professional care providers working in child and family services. WAC is a care coordination method targeting families with complex needs. The core components of WAC involve activating family members and the social network, integrating the care provider network, and assessing, planning and evaluating the care process. WAC was introduced in the Netherlands using two approaches: the network approach (NA) and the team approach (TA).

**Methods:**

A cross-sectional study was conducted using a digital questionnaire targeted at care providers. After imputation of missing data, univariate and multilevel regression analyses were conducted to study the associations between adherence to the core components of WAC, the determinants of adherence and background characteristics.

**Results:**

In total 145 out of 275 care providers (52.7%) responded to the questionnaire. Multilevel regression analysis showed that self-efficacy of the care providers and the way WAC is organised (NA versus TA region) were significantly associated with adherence to core components of WAC. Self-efficacy was significantly associated with all WAC core components (activating family members and the social network: β (95% confidence interval, CI) = .27(.04–.50), integrating the network of care providers: β (95% CI) = .27(.05–.50) and assessing, planning and evaluating the care process: β (95% CI) = .30(.08–.52)). The way WAC is organised was significantly associated to two core components (activating family members and the social network: β (95% CI) = .18(0.1–.37) and integrating the network of care providers: β (95% CI) = .25(.09–.42)).

**Conclusion:**

The way WAC is organised and the self-efficacy of care providers who use WAC are factors that are relevant for the redesign of the strategy for introducing WAC. Longitudinal research into the predictive value of determinants of adherence to WAC is advised.

**Electronic supplementary material:**

The online version of this article (10.1186/s12913-018-3774-6) contains supplementary material, which is available to authorized users.

## Background

Optimal care for families with complex needs represents a challenge for both professional care providers and families. When treating these families, care providers often find it hard to deliver well-planned and patient-centred care [7]. These challenges are linked to a mix of family problems and multi-morbidity that make it difficult to meet the specific needs and preferences of families. Wrap around care (WAC) is a method for care coordination that targets these families with complex needs who use child and family services [4, 8]. The core components of WAC are 1. activating family members and the social network, 2. integrating the care provider network and 3. assessing, planning and evaluating the care process [5, 6]. A meta-analysis of the effectiveness of WAC found that it had a positive impact on the living situation of young people, juvenile justice outcomes, mental health outcomes, school performance and the overall functioning of the child [26].

The actual impact of innovations like WAC, defined as a program perceived as new by professional care providers in their care setting, is the product of the efficacy of the method (the extent to which WAC can resolve the problems that families encounter) and the level of adherence (the extent to which WAC is implemented by all care providers and the families). Full adherence to an innovation will be unlikely in daily practice and depends on the systematic introduction of the method. Several models describe planning sequences for promoting the systematic implementation of an innovation like WAC in general terms [3, 4, 9, 11, 13, 15–17, 20, 22, 23]. The first step involves identifying and analysing the determinants that impede or enhance the use of an innovation. Secondly, strategies targeting the most important determinants need to be put in place to introduce the innovation in conjunction with standard activities such as the selection and training of care providers and the evaluation of the innovation [12, 14]. Thirdly, both care providers and clients should be studied to establish the extent to which the innovation is actually used and to examine the determinants of use in relation to the innovation strategies to which the care providers are exposed. There have been only a few analyses of the use of innovations or their determinants with a view to underpinning the systematic introduction of the intervention or method [10, 19]. The aim of this study is, therefore, to improve our understanding of the determinants of adherence to wrap-around care (WAC) by professional care providers working in child and family services. This aim corresponds to the last step of the planning sequence described here. We examine the association between the degree of adherence to WAC core principles, the relevant determinants and background characteristics.

## Methods

In this observational study, we followed the process and implementation of the in the USA developed WAC method. This method was used in two Dutch regions to organize the care for families with complex needs. These regions used a network-based approach (NA) and a team-based approach (TA) for delivering WAC. We assessed the innovation strategies using Fixsen’s framework for innovation strategies [12, 28]. Fixsen distinguishes seven innovation strategies for implementation based on commonalities in successfully implemented programs reported in literature: selection of staff, preservice training, consultation and training, staff evaluation, program evaluation, facilitative administrative support and system interventions. We assessed the innovation strategies based on policy papers, interviews with care providers and managers who were responsible for use of WAC and interviews with representatives of the regional steering committees of WAC. The quality of the innovation strategies was not assessed.

In the NA region, each professional could decide when to provide WAC to which family. Sixteen child and family services in the region employing approximately 800 professionals were responsible to implement WAC in their organisations. The different service organizations used a mix of the following innovation strategies: pre-services training, consultation and coaching, program evaluation and system interventions, i.e. interventions at executive and governance level to ensure resources required to support the care providers who were entitled to use WAC. The region did not invest in the selection of professionals who use WAC, the evaluation of these users, and facilitative administrative support during the implementation process. This region had been working with WAC for five years prior to the present study.

The TA region formed three fixed multidisciplinary teams to which families could be referred for the WAC method, consisting of in total approximately 50 professionals. Local government had the responsibility for the implementation and not the child and family services. They used several innovation strategies: staff selection, pre-services training, program evaluation, facilitative administration support consisting of a secretariat for each team and a central digital client database, and system interventions. The innovation strategies consultation and coaching on the job or staff evaluation were not used. This region had been working with WAC for two years prior to present study.

The child and family services and local government of both regions participated in a Collaborative Research Centre that conducted the study. Local government as the budget holder and the child and family service organizations decided to implement WAC several years prior to the present study as a solution for poor service provision for multiproblem families. Government and services organisations in both regions were unfamiliar with how to systematically implement innovations like WAC and participated in the present study with the aim of redesigning their innovation strategy and to improve service delivery.

The innovation strategies of the regions were developed and delivered by a team of implementation agents which consisted of policy makers from the local government and child and family services and led by a coordinator. Occasionally, external experts were put in action, for example to train professional care providers in the WAC method. These activities were mostly funded by the local government. WAC was the only method implemented in the child and family services at the time of this study.

### Participants and design

A cross-sectional design was used to collect opinions and perceptions of professional care providers on adherence to WAC principles and its determinants in 2013. A random sample of 221 (27%) of all eligible care providers (*n* = 813) working in the sixteen child and family services in the NA region were asked to fill in a digital questionnaire. All 54 care providers of the local WAC teams in the TA region were invited to participate in the survey. These care providers worked in three different organizations. The intraclass correlations of the organizations for the three core components varied between .05 and .02. Participation in the study was anonymous. The Medical Ethics Committee considered her approval for this study as not necessary under the Dutch Law (C12.041).

### Measurements

The digital questionnaire was developed in close collaboration with an expert panel of change agents involved in the implementation process: two coordinators of the implementation of WAC, two policy-officers of the local government and four care providers working with WAC. The questionnaire addressed the care provider’s self-reported adherence to the core WAC components and the determinants of adherence (see Table 2).

The three core WAC components are: 1. activating family members and the social network, 2. integrating the care provider network and 3. assessing, planning and evaluating the care process [5, 6]. Adherence to these core components was measured by asking the respondents to indicate (on a five-point Likert scale ranging from ‘none of the families’ to ‘all families’) the number of eligible families with whom they used the WAC components or principles. Adherence was defined as the degree to which the care provider used the recommended procedures and avoided procedures not considered to be advisable or acceptable [21]. A high degree of adherence to all three core components was expected to maximise the impact of WAC.

The determinants of adherence to the three core WAC components were derived from general literature on determinants of innovation [13, 14]. They came from a shortlist of 50 determinants impacting implementation of innovations [13]. This shortlist was based on a literature review on the implementation of evidence-based innovations and programmes in the field of preventive child health care and schools health programmes, and a Delphi study among implementation experts.

Time constraints were perceived as a major obstacle for the professionals’ study participation. To avoid overburdening the professionals and organizations the questionnaire had to be concise. Therefore, the experts of each region made a selection of determinants based on two criteria: 1. the anticipated impact of a determinant on adherence and 2. the determinant had to be suitable to measure via a self-report questionnaire. The experts then chose the final determinants based on consensus. The questionnaire was pre-tested which led to minor adjustments.

The respondents were asked to tick a five-point Likert scale to indicate the perceived effect of each determinant on adherence (see Table 1). The reliability of these scales ranged from satisfactory to good (see Additional file 1: Table A1 and Table A2 for the factor analysis of the adherence scale and Additional file 2: Table B.1, Table B.2 and Table B.3 for the factor analysis of the determinants). In addition, background characteristics were assessed: how WAC was organised (NA or TA), the WAC caseload (number of families using WAC in the last six months), number of years of working experience of the care provider, sector of expertise of child and family services in which the respondents worked, and the educational level of the respondents.

**Table 1:** Scales, number of items, reliability and examples of questions in the questionnaire

Scale	Number of items	Reliability (α) / correlation coefficient (r)	Example of questions, answer categories and score range
*Adherence to the core WAC components*			
Activating family members and the social network	3	α = .70	In how many of the eligible families did you evaluate the care process? never (1)- in all families (5)- does not apply here (6) (6 categories)
Integrating care provider network	5	α =. 79	In how many of the eligible families did you collaborate with the providers of care for the child? never (1)- in all families (5)- does not apply here (6) (6 categories)
Assessing, planning and evaluating the care process	5	α = .86	In how many of the eligible families did you state concrete goals? never (1)- in all families (5)- does not apply here (6) (6 categories)
*Determinants concerning the innovation*			
Relevance for the families	1	–	To what extent do you feel WAC has an added value for families? no added value (1)- considerable added value (5) (5 categories)
Procedural clarity	5	α = .74	Estimate how familiar or unfamiliar you are with the key elements of WAC very unfamiliar (1) -very familiar (5)- does not apply (6) (6 categories)
*Determinants concerning the user of the innovation*			
Self-efficacy	2	*r* = .82	To what extent are your skills adequate to work with the WAC method? completely inadequate (1)- completely adequate (5) (5 categories)
Social support	2	*r* = .68	To what extent do you feel supported by your colleagues? not supported at all (1)- very supported (5) (5 categories)
Attitude	7	α = .61	To what extent do you think the goals of the treatment should be worded so that they are understandable for the family? not important at all (1)- very important (5) (5 categories)
*Determinants concerning the organization*			
Available time and practical support	3	α = .69	To what extent do you receive adequate administrative and other types of support for organising practical issues related to WAC? completely adequate (1)- completely inadequate (5) (5 categories)
Satisfaction with WAC	1	–	To what extent are you satisfied with collaboration within WAC? completely dissatisfied (1)- completely satisfied (5) (5 categories)
*Determinants of the context*			
Legislation	1	–	To what extent does the WAC approach fit in with current legislation and regulations? very poorly (1)- very well (5) (5 categories)

### Statistical analyses

The first step in the analyses involved establishing the scales for the measurement of adherence to the core components of WAC and the determinants using principal axis factoring for non-normal and principal factor analysis for normal distributed scales, and reliability analyses. Secondly, multiple imputation was applied to adjust for missing values. This simulation-based approach created a number of imputed (completed) data sets by ‘filling in’ plausible values for the missing data. The imputations were based on a model that used information from other variables to achieve optimal estimates. Only imputations for the missing values between the lowest and highest values of the measured outcome variable were considered valid. Uncertainty about the model estimates was reflected in differences between imputations in the different completed data sets. We used multivariate imputation by chained equations to create ten imputed data sets based on general characteristics, determinants, measurements of adherence, and the WAC components [27]. We applied predictive mean matching to create multiple imputations. Confidence intervals for the outcomes were estimated through pooling results from the completed data sets [24].

Descriptive statistics were then used on the imputed data to give an overview of the characteristics of the respondents per region using t-tests or ANOVA. Total scale scores were calculated for each core adherence component and each region, with higher scores representing higher adherence to WAC. The associations between the background characteristics, the determinants and the adherence to the three core components of WAC were then tested at the univariate level using logistic regression for categorical and linear regression for continue variables. The background characteristics with a significant bivariate association and all other determinants were entered in multilevel regression models with organization as level and the WAC core components as outcome variables. Nineteen organizations were entered. All statistical analyses were performed in SPSS version 20.0 for Windows [18]. A two-tailed significance level of .05 was used in all analyses.

## Results

### Respondents and their scores for each core component

A total of 145 of the 275 care providers completed the questionnaire (52.7%), with missing data per determinant varying from none to 35.9%: 97 care providers from the NA region (43.9%) and 48 care providers (88.9%) from the TA region (see Table 2). The majority of the respondents had received higher vocational education and they worked in primary care or youth care. Significantly more respondents in the NA region were employed in mental health services than in the TA region.

**Table 2:** Characteristics of the respondents and mean scores for adherence to core WAC components by strategy (network-based or team-based)

		Network-based (*n* = 97)	Team-based (*n* = 48)	Total (*n* = 145)
*Determinants of adherence*		n (%)	n (%)	n (%)
Educational level	vocational education and training	7.2 (7.2)	6 (12.5)	13.2 (9.0)
	applied scientific and university	89.8 (92.8)	42 (87.5)	131.8 (91.0)
Sector of child and youth services	preventive child health care	19 (19.6)	10 (20.8)	29 (20.0)
	primary care	23 (23.7)	24 (50.0)	47 (32.4)
	mental health care *	29 (29.9)	3 (6.3)	32 (22.1)
	youth care	26 (26.8)	11 (22.9)	37 (25.5)
		M (SD)	M (SD)	M (SD)
Experience as care provider in child and family services in number of years		11.5 (9.3)	11.3 (8.3)	11.4 (9.0)
Caseload as care coordinator in past six months		2.7 (4.6)	2.6 (3.9)	2.6 (4.4)
*Adherence to core WAC components*		M (SD)	M (SD)	
Activating family members and the social network		2.1 (1.2)	2.5 (1.30)	
Integrating care provider network **		2.8 (1.6)	3.3 (1.6)	
Assessing, planning, and evaluating the care process **		2.6 (1.6)	3.5 (1.7)	

Values are expressed as a mean (M) and standard deviation (SD) or n (%). *p < 0.05 **p < 0.01

The care providers working in the NA region reported significantly higher scores on scales for adherence to the core components planning, assessing and evaluating the care process and integrating the care provider network than their counterparts in the TA region (see Table 2).

### Determinants of adherence to WAC components

As seen in Table 3, the determinants the way WAC is organised (NA or TA), the relevance of using WAC for the families themselves, support from colleagues and management reported by the care provider using WAC (social support), the attitude of the care provider towards WAC and the time available and practical support for using WAC were significantly associated in the univariate multilevel analyses with adherence to one or more core components. The procedural clarity of the method and the self-efficacy of the care providers using WAC were significantly associated with adherence to all core components.

**Table 3:** Multilevel regression analyses and the degree of adherence to WAC core components

Determinants	Adherence to components					
	Activating family and the social network^#^		Integrating care provider network^##^		Assessing, planning and evaluating the care process^###^	
	Crude ^a^	Adjusted ^b^	Crude ^a^	Adjusted ^b^	Crude ^a^	Adjusted ^b^
	β (95% CI)	β (95% CI)	β (95% CI)	β (95% CI)	β (95% CI)	β (95% CI)
Organisation of WAC (team-based = ref)	.16 (−.00;.32)	.18 (0.1;.37)*	.16 (−.01;.33)	.17 (−.00;.34)	.25 (.09;.40)**	.25 (.09;.42)**
Relevance for families	.15 (−.01;.31)	.03 (−1.14;.20)	.23 (.07;.40)**	.13 (−.04;.29)	.21 (.05;.37)*	.10 (−.06;.26)
Procedural clarity	.27 (.10;.44)**	.12 (−.07;.32)	.31 (.17;.48)***	.18 (−.01;.37)	.08 (.03;.13)**	14 (−.05;.33)
Self-efficacy	.25 (.09;.89)**	.27 (.04;.50)*	.29 (.14;.45)***	.27 (.05;.50)*	.27 (.11;.43)***	.30 (.08;.52)**
Social support	.14 (−.02;.30)	−.10 (−.33;.12)	.22 (.06;.38)**	−.02 (−.24;.21)	.20 (.04;.35)**	−.02 (−.24;.20)
Attitude	.22 (.06;.38)**	.14 (−.02;.31)	.14 (−.02;.30)	.04 (−.11;.20)	.13 (−.03;.29)	.04 (−.12;.20)
Available time and practical support	.18 (.01;.35)*	.05 (−.15;.25)	.14 (−.03;.30)	−.07 (−.27;.13)	.12 (−.05;.29)	−.06 (−.25;.13)
Satisfaction WAC	−.04 (−.21;.12)	−.00 (−.17;.17)	−.06 (−.23;.10)	−.04 (−.21;.12)	−.10 (−.28;.06)	−.06 (−.23;.09)
Legislation	.06 (−.10;.23)	.03 (−.14;.20)	.17 (−.24;2.38)	.12 (−.05;.28)	.15 (−.02;.30)	.12 (−.02;.31)

^#^ τ^2^ = .69 ^##^τ^2^ = 3.21 ^###^τ^2^ = 1.33. ^a^ β represents the β of the univariate multilevel regression analysis with organization as level, adherence to WAC as outcome, the determinant as independent variable. ^b^ β represents the β of the multivariate multilevel regression analysis with organization as level and adherence to WAC as outcome, the determinant as independent variables and all other determinants in the model as co-variates. *p < .05** *p* < .01 ***p < .001

In the multivariate multilevel models, the way WAC was organised and the self-efficacy of the care provider using WAC remained significantly associated with adherence to respectively two and all three core WAC components. The way WAC was organised was significantly associated with higher adherence scores for the WAC core components activating family members and the social network and assessing, planning and evaluating the care process (with NA scoring higher than TA). Higher perceived self-efficacy was associated with higher scores for activating family members and the social network, integrating the care provider network, and assessing, planning and evaluating the care process.

The results for the multilevel models on the non-imputed data were in line with the results for the imputed data: associations between self-efficacy of the care givers, the way WAC was organized and adherence to several core components of WAC were also found. On top of these determinants, attitude of the care provider towards WAC also showed a significant association to the adherence to WAC in the non-imputed data.

## Discussion

This study shows that adherence to wrap-around care (WAC) among professional care providers working in child and family services has been linked to the self-efficacy of the care providers and the way WAC is organised. The network-based approach (NA) to implementation leads to more positive results than the team-based approach (TA).

Research into adherence to WAC principles showed that adherence to the core component activating family members and the social network was relatively weak by comparison with the other two core components [25]. Another study noted the absence of support systems for families with complex needs, making it difficult for WAC teams to attain the desired adherence to the core component activating family members and the social network [8]. In these circumstances, the self-efficacy of the professional toward WAC principles may be decisive in terms of achieving the desired involvement and the activation of the families and the social network, as we found in this study. Research shows that the perceived self-efficacy of professionals is a known determinant of the implementation of innovations in health care [14]. Although implementation research looking at WAC focuses more on the organisation culture or climate, this study found that self-efficacy as perceived by the care providers is also an important determinant that should be targeted when introducing the WAC care [4, 8].

We also found that the way WAC was organized is relevant for adherence to two core components. The finding that NA leads to higher adherence than TA was not expected. A known risk of top-down and large-scale implementation processes such as those used in the NA region is that they fail to address local needs and concerns. These proven difficulties are circumvented when WAC is introduced using local teams. The two regions differed in their approaches, which possibly have suppressed the variables that were significant at univariate level. The organization of WAC may encompass these separate variables who showed to be relevant at univariate level. For example, the determinant procedural clarity was associated with all WAC core components at univariate level. However, in the multivariate model the associations of clarity with the outcomes dropped and were no longer significant. This is explained by a confounding effect of the other determinants, including the way WAC was organized.

### Strengths and limitations

A strength of this study was the wide range of experience of the respondents with WAC varying from non-existent to substantial. We also included child and youth care providers of several type of organizations in the study. The significant higher amount of care providers in mental health services in the NA region was due to the limited amount of care providers using WAC in the TA region. All care providers from mental health services of the TA region participated in this study. Non-response was higher in the NA region than in the TA region. Although we don’t know the characteristics of the non-responders because they were not systematically collected, this higher non-response could have led to an overestimation of adherence in the NA region.

A limitation was that the length of the questionnaire was reduced due to time constraints for the organizations participating in this study. More influential determinants may therefore have been missed [14]. Further development is advised to enhance the validity of the scales measuring adherence and its determinants. Nevertheless, allowing the professionals involved with the implementation of WAC to choose the determinants that they found most appropriate made it possible to adapt the questionnaire to the specific challenges faced by the regions. Another limitation was the use of self-reported adherence measures, which may result in the bias of actual adherence by comparison with methods based on objective data such as observations [1].

### Implications for further practice

Our findings imply that the self-efficacy of care providers should be at the heart of implementation strategies for WAC. Triangulation by means of several group meetings with care providers was used to establish an in-depth picture of how their self-efficacy relating to WAC can be improved. Care providers said that they did feel insecure with respect to mastering the value-based WAC method. They had no previous experience with WAC and had worked in the past only with clear guidelines or more protocolled methods. The care providers preferred learning on the job as a way of mastering working practices based on values. Modelling, which is a feature of learning on the job, is a known way of increasing self-efficacy in line with Bandura’s social cognitive theory [2].

In addition, we advise focusing on the other determinants that are significantly associated with implementation when redesigning the innovation strategy, encompassed by the way WAC was organized, i.e. the NA and TA approaches. Steps should be taken to ensure that professional care providers feel that they have the support of their colleagues and management, that they have enough time and the practical support they need to use WAC, that care providers have a positive attitude towards WAC, that they understand the relevance for the families and that the procedures for using WAC are clear. We recommend a bottom-up, team-based approach, since theory predicts that this TA-approach is most likely to lead to support and motivation for the users of the WAC method.

### Implications for further research

More research is needed to equip care providers with the methodological tools required to ensure that they have the feeling that they master WAC. Longitudinal research is recommended into the predictive value of the determinants of adherence to WAC and the effect of how WAC is organised. Testing should include not only self-reported adherence but also observations or case records of what WAC care providers actually do in practice. Recently the Team Observation Measure was developed for valid observations of use of WAC components in practice [9]. Qualitive research could give more insight in how the different ways WAC was organized affect the adherence to WAC. Finally, research is required into the effect of adherence to WAC by care providers in terms of improving family functioning.

## Conclusions

This study shows that the way WAC is organised and the self-efficacy of care providers who use WAC are significantly associated with the adherence to the core components of WAC. We advise to build on these determinants of adherence when redesigning the innovation strategy, such as developing a method for learning on the job as a way of promoting care providers’ self-efficacy. Finally, further qualitative research into the way the innovation is organized and its effect on adherence is advised as well as longitudinal research into the predictive value of determinants of adherence to WAC.

## Additional files

Additional file 1: **Table A1.** Factor analyses resulting in two adherence core components: ‘assessing, planning and evaluating the care process’ and ‘activating family and their social network’. **Table A2.** Factor analysis resulting in the adherence core component ‘integrating care provider network’. (DOCX 38 kb)

Additional file 2: **Table B.1.** Factor analysis resulting in the determinant ‘procedural clarity’. **Table B.2**. Factor analysis resulting in the determinant ‘available time and practical support’. **Table B.3**. Factor analysis resulting in the determinant ‘attitude’. (DOCX 39 kb)

## Abbreviations

NA: Network approach
TA: Team-based approach
WAC: Wrap around care

## References

[1] Adams AS, Soumerai SB, Lomas J, Ross-Degnan D (1999). Evidence of self-report bias in assessing adherence to guidelines. Int J Qual Health Care.

[2] Bandura A (1977). Self-efficacy: toward a unifying theory of behavioral change. Psychol Rev.

[3] Bartholomew LK, Parcel GS, Kok G, Gottlieb NH, Fernandez ME (2011). Planning health promotion programs: an intervention mapping approach.

[4] Bertram RM, Suter JC, Bruns EJ, O'Rourke KE. Implementation research and wraparound literature: building a research agenda. J Child Fam Stud. Dec 2011;20(6):713–25.

[5] Bruns EJ, Burchard JD, Suter JC, Leverentz BK, Force MM (2004). Assessing Fidelity to a community-based treatment for youth: the wraparound Fidelity index. J Emot Behav Disord Sum.

[6] Bruns EJ, Suter JC, Force MM, Burchard JD (2005). Adherence to wraparound principles and association with outcomes. J Child Fam Stud.

[7] Bruns EJ, Burchard JD, Yoe JT (1995). Evaluating the Vermont system of care: outcomes associated with community-based wraparound services. J Child Fam Stud.

[8] Bruns EJ, Pullmann MD, Sather A, et al. Effectiveness of wraparound versus case Management for Children and Adolescents: results of a randomized study. Adm Policy Ment Health. 2015;42:309. 10.1007/s10488-014-0571-3. 10.1007/s10488-014-0571-3 PMC4278946 24973891

[9] Bruns EJ, Weathers ES, Suter JC, Hensley S, Pullmann MD, Sather A. Psychometrics, reliability, and validity of a wraparound team observation measure. J Child Fam Stud. 2014;24(4):979-91. 10.1007/s10826-014-9908-5 PMC4465793 26085783

[10] Colquhoun HL, Squires JE, Kolehmainen N, Fraser C, Grimshaw JM (2017). Methods for designing interventions to change healthcare professionals' behaviour: a systematic review. Implement Sci.

[11] Feldstein AC, Glasgow RE (2008). A practical, robust implementation and sustainability model (PRISM) for integrating research findings into practice. Jt Comm J Qual Patient Saf.

[12] Fixsen DL, Blase KA, Naoom SF, Wallace F (2009). Core implementation components. Res Soc Work Pract.

[13] Fleuren M, Wiefferink K, Paulussen T (2004). Determinants of innovation within health care organizations. literature review and delphi study. Int J Qual Health Care.

[14] Fleuren MA, Paulussen TG, Van Dommelen P, Van Buuren S (2014). Towards a measurement instrument for determinants of innovations. Int J Qual Health Care.

[15] Glasgow RE, Vogt TM, Boles SM (1999). Evaluating the public health impact of health promotion interventions: the RE-AIM framework. Am J Public Health.

[16] Greenhalgh T, Robert G, Macfarlane F, Bate P, Kyriakidou O (2004). Diffusion of innovations in service organizations: systematic review and recommendations. Milbank Q.

[17] Grol R, Grimshaw J (2003). From best evidence to best practice: effective implementation of change in patients' care. Lancet.

[18] IBM corp (2011). IBM SPSS statistics for windows 20.0.

[19] Liang, L., Bernhardsson, S., Vernooij, R.W.M., Armstrong, M.J., Bussières, A., Brouwers, M.C. & Gagliardi, A.R. (2017). Use of theory to plan or evaluate guideline implementation among physicians: a scoping review. Implementation Science201712:26, 12:26 doi: 10.1186/s13012-017-0557-0. 10.1186/s13012-017-0557-0 PMC5327520 28241771

[20] Michie S, Fixsen D, Grimshaw JM, Eccles MP (2009). Specifying and reporting complex behaviour change interventions: the need for a scientific method. Implement Sci.

[21] Perepletchikova F, Treat TA, Kazdin AE (2007). Treatment integrity in psychotherapy research: analysis of the studies and examination of the associated factors. J Consult Clin Psychol.

[22] Powell BJ, Proctor EK, Glass JE (2014). A systematic review of strategies for implementing empirically supported mental health interventions. Res Soc Work Pract.

[23] Proctor EK, Landsverk J, Aarons G, Chambers D, Glisson C, Mittman B (2009). Implementation research in mental health services: an emerging science with conceptual, methodological, and training challenges. Adm Policy Ment Health Ment Health Serv Res.

[24] Rubin DB. Multiple imputation for nonresponse in surveys. New York: Wiley; 1987.

[25] Snyder EH, Lawrence CN, Dodge KA (2012). The impact of system of care support in adherence to wraparound principles in child and family teams in child welfare in North Carolina. Child Youth Serv Rev.

[26] Suter JC, Bruns EJ (2009). Effectiveness of the wraparound process for children with emotional and behavioral disorders: a meta-analysis. Clin Child Fam Psychol Rev.

[27] van Buuren S (2012). Flexible imputation of missing data.

[28] Wells R, Gifford EJ. Implementing a case management initiative in high-need schools. Child Youth Serv Rev. 2013;35(5):787–96. 10.1016/j.childyouth.2013.01.026 PMC3747008 23976809

