# Supplementary material for: Determinants of adherence to wrap-around care in child and family services
Source: BMC Health Serv Res. 2019 Jan 28;19:76. doi: 10.1186/s12913-018-3774-6 (PMC6350391; doi:10.1186/s12913-018-3774-6)
Supplement: Supplementary file 1 — Table A1. Factor analyses resulting in two adherence core components: ‘assessing, planning and evaluating the care process’ and ‘activating family and their social network’. Table A2. Factor analysis resulting in the adherence core component ‘integrating care provider network’. (DOCX 38 kb) [file 12913_2018_3774_MOESM1_ESM.docx]

**Additional file A Factor analyses of items regarding the outcome adherence**

**Table A1** Factor analyses resulting in two adherence core components: ‘assessing, planning and evaluating the care process’ and ‘activating family and their social network’^a^

| Question | Assessing, planning and evaluating the care process^1^ | Activating family  and their social network^2^ |
| --- | --- | --- |
| In how many of the eligible families did you had one treatment plan for the entire family? | .59 | .24 |
| In how many of the eligible families did the family have access to the treatment plan? | .76 | .13 |
| In how many of the eligible families did you formulate the goals of the treatment in the way that they were understandable for the family? | .66 | .38 |
| In how many of the eligible families did you state concrete goals? | .83 | .21 |
| In how many of the eligible families did you plan regular moments for evaluation of the care process? | .77 | .20 |
| To what extent are goals stated which could only be obtained with the help of the support network of the family? | .12 | .99 |
| To what extent are members of the social support network of the family present at treatment sessions? | .29 | .49 |
| In how many of the eligible families did you state goals which were to be pursued by the family themselves? | .58 | .47 |

^a^ Extraction Method: Principal Component Analysis, Rotation Method: Varimax with Kaiser Normalization; ^1^α=.88; ^2^α=.70

**Table A2** Factor analysis resulting in the adherence core component ‘integrating care provider network’^a^

| Question | Integrating care provider network^1^ |
| --- | --- |
| In how many of the eligible families did you assess the financial situation of the family? | .75 |
| In how many of the eligible families did you collaborate with the providers of care for the child? | .56 |
| In how many of the eligible families did you collaborate with the school for the child? | .43 |
| In how many of the eligible families did you collaborate with the care providers for the parent(s)? | .91 |
| In how many of the eligible families did you collaborate with service providers? | .65 |

^a^ Extraction Method: Principal Component Analysis, Rotation Method: Varimax with Kaiser Normalization; ^1^α =.70
