# Supplementary material for: Determinants of adherence to wrap-around care in child and family services
Source: BMC Health Serv Res. 2019 Jan 28;19:76. doi: 10.1186/s12913-018-3774-6 (PMC6350391; doi:10.1186/s12913-018-3774-6)
Supplement: Supplementary file 2 — Table B.1. Factor analysis resulting in the determinant ‘procedural clarity’. Table B.2. Factor analysis resulting in the determinant ‘available time and practical support’. Table B.3. Factor analysis resulting in the determinant ‘attitude’. (DOCX 39 kb) [file 12913_2018_3774_MOESM2_ESM.docx]

**Additional file B Factor analyses of items regarding the determinants**

**Table B.1** Factor analysis resulting in the determinant ‘procedural clarity’ ^a, 1^

| Question | Factor |
| --- | --- |
| Estimate how familiar or unfamiliar you are with the key elements of WAC | .75 |
| Estimate how familiar or unfamiliar you are with the target families of WAC | .75 |
| Estimate how familiar or unfamiliar you are with where you can find information about WAC and its p procedures? | .77 |
| Estimate how familiar or unfamiliar you are with the procedures of other child and family services w working with WAC in the region? | .56 |
| Estimate how familiar or unfamiliar you are with the procedure for escalating if there are issues in the c continuation of the care process, for example because of waiting lists? | .58 |

^a^ Extraction Method: Principal Component Analysis, Rotation Method: Varimax with Kaiser Normalization. ^1^α =.74

**Table B.2** Factor analysis resulting in the determinant ‘available time and practical support’ ^a, 1^

| Question | Factor |
| --- | --- |
| To what extent do you have time to work with WAC? | .85 |
| To what extent do you receive adequate administrative and other types of support for organising practical issues related to WAC? | .75 |
| To what extent are procedures in your own organization hindering you when working with WAC? | .77 |

^a^ Extraction Method: Principal Component Analysis, Rotation Method: Varimax with Kaiser Normalization. ^1^α =.69

**Table B.3** Factor analysis resulting in the determinant ‘attitude’ ^a, 1^

| Question | Factor |
| --- | --- |
| To what extent do you think families should have access to the treatment plan? | .44 |
| To what extent do you think the goals of the treatment should be worded so that they are und understandable for the family? | .37 |
| To what extent do you think goals should be formulated in a way that they could be pursued by the the family themselves? | .40 |
| To what extent do you think goals should be formulated in a way in which they could be obtxobtained with help of the social support network of the family? | .44 |
| To what extent do you think the social support network should be present at treatment ssssssessions? | .42 |
| To what extent do you think families should have one treatment plan for the entire family? | .42 |
| To what extent do you think the treatment plan should entail the goals and actions of care ppppproviders of all family members? | .47 |

^a^ Extraction Method: Principal Component Analysis, Rotation Method: Varimax with Kaiser Normalization. ^1^α =.61
